# Mitigating Portland Cement CO_2_ Emissions Using Alkali-Activated Materials: System Dynamics Model

**DOI:** 10.3390/ma13204685

**Published:** 2020-10-21

**Authors:** Moncef L. Nehdi, Abdallah Yassine

**Affiliations:** Department of Civil and Environmental Engineering, Western University, London, ON N6A 5B9, Canada; ayassine@uwo.ca

**Keywords:** CO_2_ emission, system dynamics, model, policy, portland cement, climate change, alkali-activated

## Abstract

While alkali-activated materials (AAMs) have been hailed as a very promising solution to mitigate colossal CO_2_ emissions from world portland cement production, there is lack of robust models that can demonstrate this claim. This paper pioneers a novel system dynamics model that captures the system complexity of this problem and addresses it in a holistic manner. This paper reports on this object-oriented modeling paradigm to develop a cogent prognostic model for predicting CO_2_ emissions from cement production. The model accounts for the type of AAM precursor and activator, the service life of concrete structures, carbonation of concrete, AAM market share, and policy implementation period. Using the new model developed in this study, strategies for reducing CO_2_ emissions from cement production have been identified, and future challenges facing wider AAM implementation have been outlined. The novelty of the model consists in its ability to consider the CO_2_ emission problem as a system of systems, treating it in a holistic manner, and allowing the user to test diverse policy scenarios, with inherent flexibility and modular architecture. The practical relevance of the model is that it facilitates the decision-making process and policy making regarding the use of AAMs to mitigate CO_2_ emissions from cement production at low computational cost.

## 1. Introduction

The global average of temperature has risen by about 0.85 °C from the year 1880 to 2012 [1]. The World Meteorological Organization [2] warns that greenhouse gas (GHG) concentrations are at record levels and if the current trend continues, we may see temperature increases of 3–5 °C by the end of the century, overshooting the targeted goal of 2 °C stated in the Paris Agreement. This is expected to result in higher sea levels (24 to 30 cm in year 2065 above its level in 2019) due to the melting of arctic ice, warming of ocean temperature, and more extreme weather events inflicting increased damage to civil infrastructure, living species, crops and ecosystems [3]. In addition, it is expected that rainforests will experience dry-ups, mountain glaciers will melt, and global freshwater supply will decease dramatically.

The above global challenges are related to increased greenhouse gas (GHG) emissions leading to climate change. The concentration of GHGs in the atmosphere has amplified since the industrial revolution and is projected to maintain its rise in the foreseen future. The most abundant GHG is carbon dioxide (CO_2_), representing about two thirds of all GHG emissions. According to NASA [4], CO_2_ levels are at their highest in 650,000 years, presently reaching 411 parts per million (ppm). The atmospheric concentration of CO_2_ in the 19th century was only about 280 ppm. CO_2_ emission has increased primarily due to combustion of fossil fuels for electricity and heat production, industrial processes, transportation, farming and land use and deforestation.

The cement industry alone emits about 9% of global CO_2_ emissions [5]. If it were a country, this industry would rank third after China and the USA in total GHG emissions. With urbanization and world population projected to increase to about 9.8 billion people by year 2050, the production of concrete is not expected to diminish, and cement will continue to be the world’s second most consumed commodity after water. According to the UN [3], the global urban population is expected to grow from 55% in 2018 to 68% by 2050, resulting in larger projected concrete consumption. Accordingly, the production of Ordinary Portland Cement (OPC) will increase to about 6 billion tons over the same period. In account of this colossal challenge, the cement industry has been making sustained investments and efforts to reduce its environmental footprint and enhance the sustainability record of cement production.

The production of OPC clinker is done at about 1450 °C and is therefore energy intensive. It involves calcination of limestone, which produces calcium oxide and releases CO_2_ into the atmosphere. The production of one ton of cement clinker releases about one ton of CO_2_, half of which is due to calcination of limestone and the other half is derived from fuel-based energy consumption. Some researchers [5,6] explored alternative technologies for reducing CO_2_ emissions from OPC production using blended cements. This resulted in little effect on CO_2_ emissions, except for using blended cements. Yet, blended cements still contain a major portion of OPC, which stimulated research for alternative binders with lower GHG emissions.

Alumino-silicate based materials have emerged as a very promising alternative binder to OPC since they typically consume less energy and require no heat-intensive calcination. Alkali activated materials (AAMs) (also called in this text alkali activated binders “AABs”) consist of a precursor, an activator, and water. The precursor can be an industrial by-product of a heat-intensive calcination process, such as ground granulated blast furnace slag and fly ash, or natural processed minerals like metakaolin. The activator is usually an alkaline solution comprised of hydroxide or silicate solution, depending on the precursor and type of application.

Most research on the sustainability of AAMs used Life Cycle Assessment (LCA) methodologies. For instance, [7,8] proposed a “cradle to gate” model, accounting for extraction of base minerals, transportation to production plants, and emissions released at the production stage of OPC and AAM concrete. Yet, the LCA methodology examines a fragmented sector of a complex system of systems. Since the “cradle to gate” model does not capture the environmental impact beyond the gate, the approach is only useful when comparing the production of an AAM to normal OPC.

The input data used to conduct LCA simulations is generally a fixed database built in analysis software and is not time dependent. The methodology utilizes the fixed input variables to simulate a life-cycle assessment in a linear manner without considering any feedback from the original source of data. Input variables that vary over time, such as repair and replacement, service life performance, and ageing of concrete structures, can play an important role in capturing the system’s complex behavior, but are unfortunately not exhibited in the input database of a typical LCA software. The LCA approach also lacks flexibility in accommodating emerging information and evolving scenarios, such as changes in the projected consumption of cement resulting in feedback that influences other factors of the system. Hence, non-linear time-dependent feedback loops affecting the system behavior are not adequately considered.

A suitable methodology for evaluating the sustainability of alternative binders can be based on a systems-thinking approach. This can offer a robust tool for policymaking and testing various probable scenarios. In the present study, a System Dynamics (SD) model was created to simulate CO_2_ emissions of the cement industry over the next thirty years. The model uses feedback resulting from a varying market share of OPC due to increased market share of AAMs. The effects of the type of precursor, type of activator, AAM market share and policy implementation period are integral parts of this model. The ageing, service life performance, repair and replacement of structures incorporating AAM, and sequestering of atmospheric CO_2_ via carbonation, are included to capture the time-dependent feedback loops embedded in the sustainability of these binders. The system dynamics basis of the model, model design, simulations and simulation results are discussed below.

## 2. Objectives, Novelty, and Research Significance

Cement production emits about 8–10% of global CO_2_ emissions. Alkali-activated materials have emerged as a highly promising alternative to reduce such CO_2_ emissions. However, there is no existing model that captures this effect. The objectives of this study are to develop a model that considers the ton CO_2_ emissions in their entire complexity as a system of systems including their time dependent nature. It is intended to develop a policy testing tool that can be used to assess diverse possible scenarios in the AAM replacement for cement and its impact on ton CO_2_ emissions.

The novelty of this work and its contribution to the state of the art lie in the fact that this is the first study that deploys system dynamics modeling to model the effect of AAM on CO_2_ emissions from cement production. It offers the first model of its kind to treat this problem in its holistic nature. In practice, the model can easily be used by any stakeholder to test policy scenarios whereby the user has full control over the inputs of the model, getting fully transparent representation in an explicit graphic and numerical format, describing the anticipated impact of the tested scenario on global CO_2_ emissions from cement production.

Countries with growing economies and large populations, such as China and India, continue to lead the production of OPC with a predicted cumulative total of 9.31- and 6.95-billion-ton CO_2_ emissions, respectively [9]. Although both countries have an abundant resource of OPC due to its constant use, the AAM production of precursors, such as fly ash and ground granulated blast furnace slag, has proven to exceed its demand and utilization in the past ten years (Making Concrete Change, 2018). Therefore, regulation in the production and consumption of such alkali-activated materials is necessary to lead a reduction in CO_2_ emissions. Despite the growing production of OPC, significant GHG emission reductions by this industry are possible and can help to reach the targets of the Paris Agreement. Several countries started implementing strategies for reducing CO_2_ emissions, such as carbon taxes. For instance, Canada has recently issued a coordinated nation-wide carbon tax. For jurisdictions using a price-based system, the carbon price will start at a minimum of $10 per ton in 2018 and rise by $10 per year to $50 per ton in 2022. Provinces with a cap-and-trade system shall have a 2030 emissions reduction target equal to or greater than Canada’s 30% reduction goal [10]. Accordingly, the systems thinking model for cement CO_2_ emissions developed in this study can be used in policy testing to assist this sector in meeting its emission reduction targets. The model departs from existing approaches that do not capture the complexity and time-dependent feedback loops of this system.

## 3. Systems Thinking Approach

### 3.1. System Dynamics

**Figure 1:**
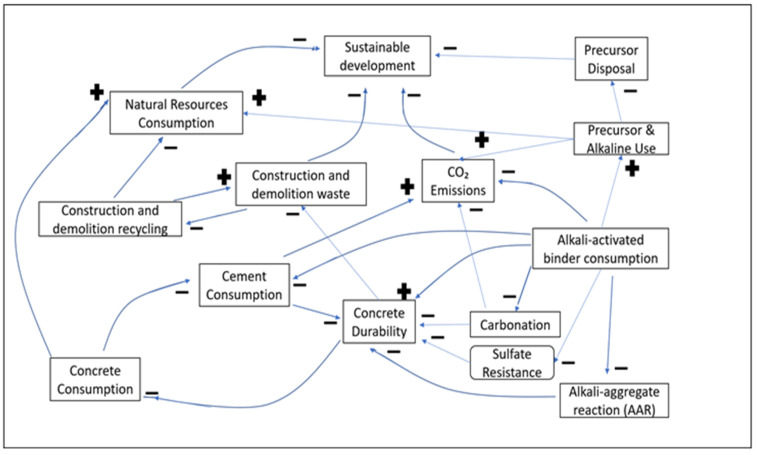
Casual Loop Diagram.

System dynamics (SD) is an object-oriented feedback-based modeling paradigm, rooted in the work of Forrester at the MIT School of Business [11]. It has been deployed in modeling complex non-linear systems, which are adaptive, counterintuitive, or governed by feedbacks. SD focuses on multiple patterns and wholes rather than static snapshots, and on “interrelationships” rather than “linear correlations”. Hence, it is widely used in businesses, education and healthcare. In SD, the qualitative relationships among the various parameters influencing a system are represented through a Causal Loop Diagram (Figure 1) or Influence Diagram [12]. The arrowhead in this figure indicates the direction of variable dependency. The negative or positive effect of a variable is set via the loop polarity using a minus (−) or plus (+) sign, respectively. A negative sign means that increasing one variable decreases the dependent variable. A positive sign insinuates that increasing the parameter increases the dependent variable and vice versa. Different types of feedback are outlined below.

### 3.2. Open and Closed Loop Systems

**Figure 2:**
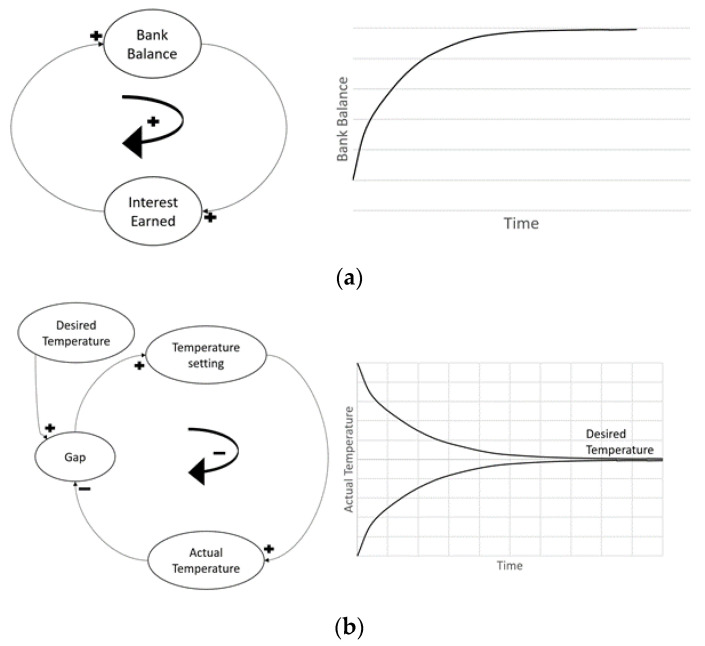
(**a**) Open Loop System; (**b**) Closed Loop System.

**Figure 3:**
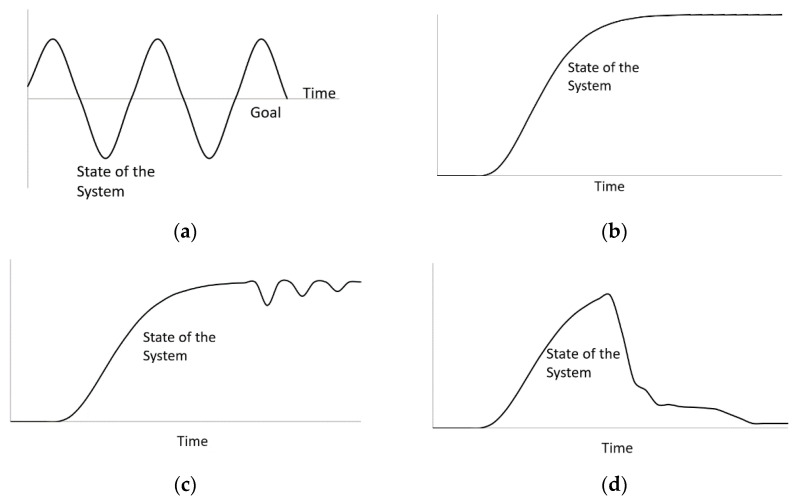
(**a**) Oscillation; (**b**) S-shaped growth; (**c**) S-shaped growth with overshoot, and (**d**) Overshoot and collapse.

Open systems do not incorporate feedback and are usually exponential functions. The system carries out the components’ actions without prediction of further consequences. For example, there are no factors that resist bacteria population growth in a moist environment; the population is rather increasing with no awareness of the system performance. The outputs of an open system are simply characterized by its inputs. Conversely, a closed system, also known as a feedback system, is aware of its performance and modifies its components to provide optimal output. For example, a dam with automatic gate sensors measures the performance or feedback of its internal components (e.g., inflow) to control and adjust other dependent components (e.g., gate width) to achieve a goal (e.g., compensating for the outer flow). Open systems are usually consistent with the type of sign displayed. An open loop system can only contain either positive or negative signs, not the co-existence of both. Combinations of both signs are used in closed loop systems because the positive and negative feedbacks serve crucial roles in expressing a goal-seeking behavior by implementing a balance, whereas an open loop system expresses an exponential growth behavior, as observed in Figure 2. A closed system with delays in timing presents a special case called oscillation. Oscillation occurs when the system reaches its goal by over-adjusting or overshooting its components, triggering a new component to reverse its actions, causing a delay in reaching its initial goal once again (Figure 3a).

### 3.3. Special Systems

Complex systems can originate from the encounter of external influences. Such feedback structures include S-shaped growth, S-shaped growth with overshoot, and overshoot and collapse (Figure 3b–d). S-shaped growth occurs when the system experiences an extraordinary amount of positive feedback, and eventually becomes constrained with negative feedback after experiencing an exponential growth. The system exhibits an S-shaped function that ultimately reaches equilibrium. When a negative feedback experiences interruption of its purpose, it will overshoot nearing the beginning of its inception. The overshoot can cause some delays in achieving the end goal of the system; hence, the system structure is an S-shaped growth with overshoot. On some occasions, the system would not be able to recover from an excessive amount of positive feedback, resulting in collapse, which is called overshoot and collapse. Most engineering systems are more complex and dynamic than linear or open systems. Accordingly, System Dynamics has been implemented in diverse fields of civil engineering, such as water resource management, construction management, infrastructure planning and development, etc. However, deploying system dynamics modeling for the sustainability of alkali-activated materials has not been accessible in the open literature.

## 4. Model Background

### 4.1. AAM Composition

To create eco-efficient AAMS, the entire process including the type of precursor and activator, production, curing and placement techniques, carbonation, aging and life cycle performance must be considered. In this study, different CO_2_ emission outcomes were explored by proportioning a variety of precursors and alkaline activators that correspond to robust single and binary AAM system cases, with various market shares and policy implementation periods. A SD model was built in a Vensim Personal Learning Edition (PLE) environment [13] and used to project CO_2_ emissions over the next thirty years.

### 4.2. AAM Precursors

Precursors commonly used for making AAMs include metakaolin (MK), fly ash (FA), and ground granulated blast-furnace slag (GGBS). MK results from calcination of kaolinite clay. Conversely, FA is a by-product of coal-fired electric power plants, which contribute to about 26% of global CO_2_ emissions [14]. The production of one ton of MK releases 450 kg of CO_2_, while one ton of FA is associated with 600 kg of CO_2_ emission [15,16]. Accordingly, many countries are in the process of discontinuing coal fired power plants. MK and Class F fly ash have low calcium content and result in the production of sodium aluminosilicate hydrate (N–A–S–H) gels with secondary zeolite crystals activated by alkaline solutions, with unique resistance to acids and high temperature [17]. GGBS is a by-product collected from iron blast furnaces. This process results in 270 kg of CO_2_ per ton of GGBS [15]. GGBS contains abundant calcium (Ca), which is involved in forming C-A-S-H gels and secondary products of hydrotalcite. Generally, AAM precursors consist of Ca, Mg, Si, Al, and Na ions to achieve co-existence of N–A–S–H and calcium aluminate silicate hydrate(C–A–S–H) gels and maximize concrete durability. Such precursors in proportionate amounts could yield desired environmental, economic, and sustainability performance [17].

### 4.3. Alkaline Activators

Alkaline solutions used in AAMs are necessary to activate precursors. The reaction dissolves precursor particles, such as monomeric units and Si-O-Si and Al-O-Al bonds. These ions are then rearranged, condensed and re-solidified to create alumino-silicate gels, depending on the calcium content. Each alkaline solution has a specific role in activating precursors, and is dependent on the amount of water supplied, the pH level of the activator, and the condition of the solution. The most common alkaline solutions used to produce AAMs are sodium hydroxide (NaOH) and sodium silicate (Na_2_SiO_3_). Potassium hydroxide (KOH) and calcium hydroxide (Ca (OH)_2_) can also be used in activating precursors for producing AAM but are less commonly used. KOH and NaOH are produced by electrolysis of water, which is energy intensive. The amount of CO_2_ produced from 1 ton of NaOH and 1 ton of KOH is 1.1 and 1.94 ton, respectively [18]. NaOH is generally preferred over KOH since it is easier to produce and nearly three times less costly. Calcium hydroxide (Ca (OH)_2_) is an alternative alkaline activator produced by calcining limestone. Producing one ton of Ca (OH)_2_ releases 740 kg of CO_2_. Alkali silicates are a product of melting carbonate salts and silica. About 1.64 ton of CO_2_ is released by one ton of Na_2_SiO_3_ [19]. The amount of alkaline activator required in AAM production varies with the ratio of Si to Al ions of the precursor.

### 4.4. Durability, Service Life and Carbonation

AAM concrete can be more durable than normal OPC concrete, which can reduce the repair and replacement of aging structures [20,21]. Provis [20] indicated that AAM can reduce the duration of carbonation depending on pH changes of the alkaline solution used. When Ca(OH)_2_ is the alkaline activator, carbonation can be prominent compared to when NaOH is used. Behfarnia and Rostami [22] indicated that the proportion of alkaline solution to precursor plays a role in the carbonation of AAM. Other parameters, such as the curing conditions and concentration of alkaline activator, also affect the carbonation depth of AAM concrete. Bernal et al. [23] questioned using aggressive accelerated carbonation testing of AAM. They insinuated that carbonation in a natural setting is generally less than 5 mm in OPC concrete and 7 mm in AAM concrete over 150 days, while accelerated tests indicate carbonation of 10 and 40 mm for OPC and AAM concrete, respectively. In the present study, sensitivity analysis of the effects of AAM carbonation and service life is conducted using a sliding input device featured in the Vensim model, which allows the user to test various AAM carbonation rates (0, 5, 10, 15, and 20%) and AAM service lifespans (50, 75, 100, 125, and 150 years).

### 4.5. Projected Cement Demand Scenarios

**Figure 4:**
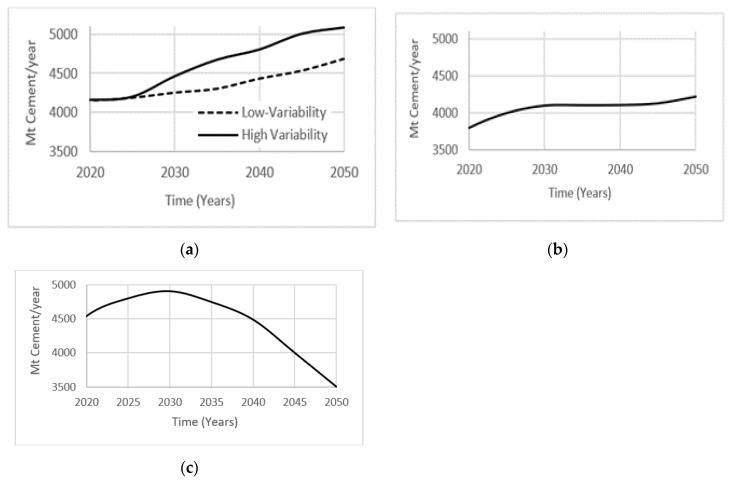
Projected cement projection based on (**a**) International Energy Agency (IEA); (**b**) carbon tax increase; and (**c**) closing GDP.

The International Energy Agency [24] projected two cases of cement consumption over the next 30 years using current patterns in gross domestic product (GDP) growth, per capita income, urbanization, growing world population, and infrastructure development needs. Depending on the assumed level of variability within the industry, cement production is expected to increase by 12–23% by the year 2050. The low variability case is implemented as a benchmark, while the high variability case is the result of sensitivity analysis performed on the production of cement demand over time in different regions (Figure 4a). Ruijven et al. [25] projected cement consumption assuming a 4% increase in carbon tax per year for the next 30 years. They predicted stabilization in cement consumption by the year 2050, as industries will defer away from manufacturing CO_2_ emission intensive products (Figure 4b). Farfan et al. [26] examined an alternate view of projected cement consumption, with closing gaps in the world’s GDP growth and infrastructure development needs. An increase in the development of African, Asian, and Latin American countries will result in a peak of global cement production around year 2030, followed by a decrease until year 2050 (Figure 4c). These diverse projected cement demand scenarios will be tested in the system dynamics model proposed in the present study.

## 5. Model Design and Architecture

**Figure 5:**
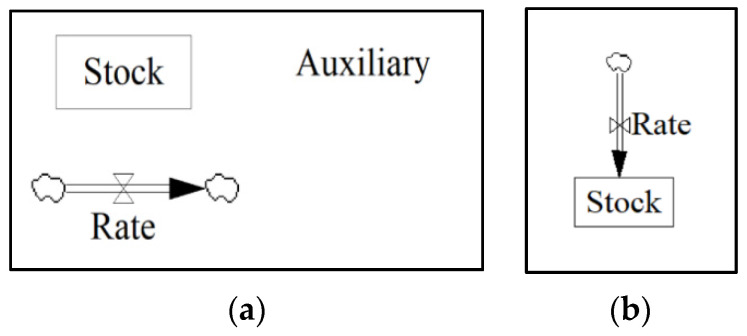
(**a**) Basic building blocks of System Dynamic modelling; and (**b**) rate flowing into a stock.

The SD model was constructed in a Vensim environment using building blocks including stocks, rates, and auxiliaries (Figure 5a). Stocks represent an accumulated amount of material usually received through a flow. A flow or rate is as an input/output function that fulfills its purpose in an instantaneous approach. Figure 5b illustrates the concept of a rate flowing into a stock that accumulates after a certain period. Auxiliaries are variables that can perform mathematical or discrete equations or can be constants. Finally, the arrows are used to develop connections between elements of the model. The Vensim model comprises the following sectors: (1) Forecast, (2) OPC and AAM Concrete, (3) Carbonation, (4) CO_2_ Emissions, and (5) AAM Composition.

**Figure 6:**
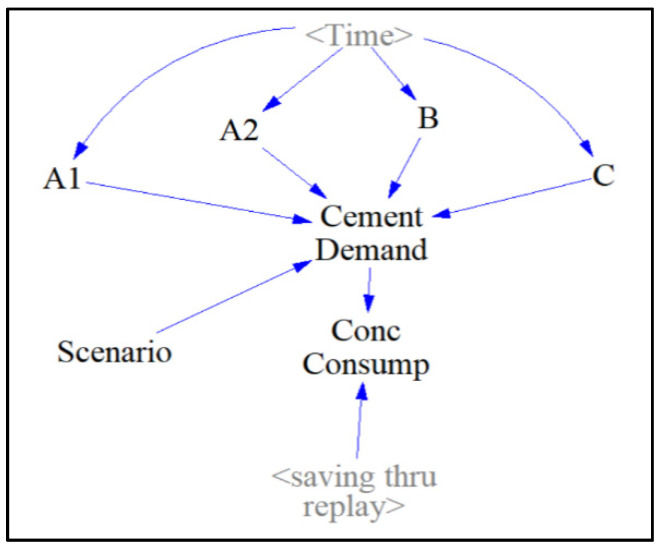
Forecast sector.

The “Forecast” sector (Figure 6) contains auxiliary components where all four projected cement demand scenarios are displayed as a function with the auxiliary. “<Time>” and the projected cement consumption data from Figure 6 are used to model each function in each auxiliary by manually inputting the amount of cement produced yearly. The user can choose which of the “Cement Demand” scenarios to examine via the sliding input device called “Scenario”. The cement demand scenarios, “A1” and “A2” represent low and high-variability projected cases of cement consumption from the IEA. Scenario “B” represents the projected case of cement consumption with a gradual increase in carbon-tax. Scenario “C” represents the projected case of cement consumption with a population stabilization after a peak of global cement production has been achieved in the year 2030. “Conc Consump” (which stands for concrete consumption), is then computed by dividing the projected cement consumption by 0.135, the assumed binder percentage composition of concrete, followed by subtracting a variable accounting for the amount of concrete consumption saved through an extended service life of concrete structures called “saving_thru_replay”. The function expression behind this variable will be further explained in the “OPC and AAB Concrete” sector.

**Figure 7:**
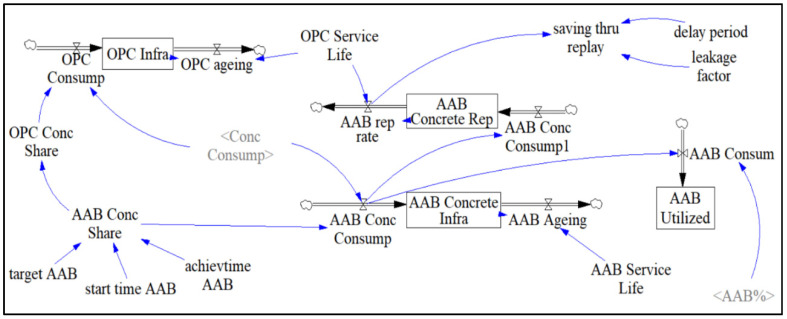
AAB and OPC Concrete Sector.

Figure 7 illustrates the “OPC and AAB Concrete” sector, which assists in estimating the amount of manufactured concrete considering factors such as service life, AAB replacement rate, and ageing of concrete structures. The targeted amount of AAB used to replace OPC can also be adjusted in this sector by using the sliding input device called “target AAB”. Scrivener et al. [14] stated that the projected market share of AABs should be within the range of 7.5–15%. Since the present study deals with policymaking, higher projected market shares of 22.5 and 30% were also tested.

**Figure 8:**
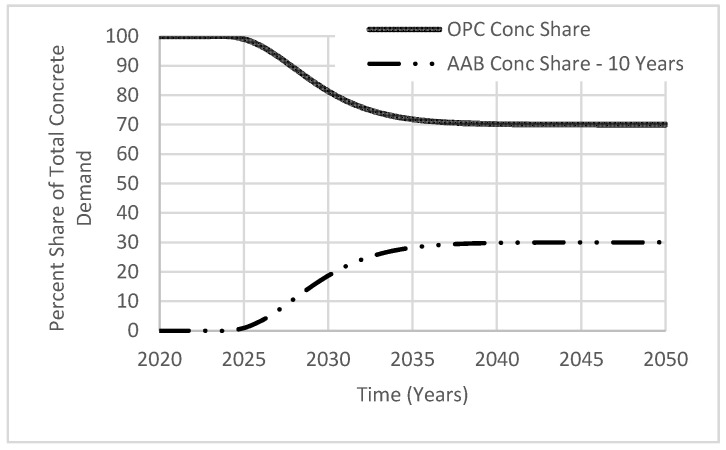
Total market share demand.

In addition to flexibility in adjusting the targeted AAB market share, the policy implementation period can also be varied with 5-year increments using the variable called “achievtime AAB”. A 10–20-year range was selected considering the simulation period (year 2020 to 2050). Once most concrete manufacturers adopt AABs, growth of its market share, “AAB Conc Share”, will resemble the shape of the letter S (Figure 8). S-shaped growth indicates the targeted goal being achieved by increased interest in AAB for enhancing concrete durability and reducing CO_2_ emissions. While the use of AAB continues to grow, the market share of OPC, “OPC Conc Share”, will continue to decline in an inverted S-shape. From the figure shown, both variables are proportional to each other as the percentage of “OPC Conc share” is calculated by subtracting the percentage of “AAB Conc Share” from 100%. The stabilization of the market share demand indicates a stabilization of future investments, as markets would reach a limit in providing materials at reasonable prices.

By multiplying the general concrete consumption computed in the forecast sector, “Conc Consump”, with the percentage share of concrete type, “OPC Conc Share”, “AAB Conc Share”, the amount of concrete consumed in each case, “OPC Consump” and “AAB Conc Consump”, shall be calculated, respectively. These variables now act as rates that flow into their respective stocks named “OPC Infrastructure” and “AAB Infrastructure”. “OPC Ageing” and “AAB Ageing” are flows draining their respective stock. The stock represents an accumulated amount of OPC concrete that is later drained using its service life, “OPC Service Life” and “AAB Service Life”, as a time variable in a Vensim embedded function called DELAY. The DELAY function acts as a conveyor carrying stock, such as the amount of OPC concrete produced during the simulation period, over a user-specified transit time, “OPC Service Life”, to fully capture the effect of ageing OPC or AAB concrete. The expression used to calculate the ageing of structures is described as:

= DELAY3(OPC Infra, OPC Service Life)

The transit time, “OPC Service Life”, can also be used to model the replacement rate of AAB concrete structures by using the DELAY function. The stock in this case represents the total amount of AAB concrete used to replace OPC concrete over the simulation time period, “AAB Concrete Rep”, which is supplied by the inflow, “AAB Conc Consump1”. The AAB concrete replacement rate shall be implemented as a factor in determining the amount of concrete consumed per year. The auxiliary variable, “saving_thru_replay”, uses the SMOOTH function to incorporate factors such as an assumed 15-year delay period of concrete structures reaching the end of their service life waiting to be replaced or repaired, AAB concrete replacement rate, and an assumed leakage factor of 0.2 to compute a more accurate result in projected concrete consumption in the following form [12]:

= SMOOTH N((1-leakage factor)*AAB rep rate, delay period, 0.2)

The amount of AAB concrete consumed each year shall also be used to calculate the amount of binder itself, “AAB Consum”, by multiplying the input variable “AAB Conc Consump” by 13.5% and “AAB%”, representing the binder composition of concrete. “AAB Utilized” acts as a stock that captures the amount of AAB utilized over 30 years by using “AAB Consum” as an inflow.

The “Carbonation” and “CO_2_ emission” sectors both depend on the amount of precursor and activator solutions consumed annually. The CO_2_ emission rate is calculated by multiplying the amount of precursor/activator consumed with the amount of CO_2_ released from one ton of the corresponding amount of precursor/activator. The rate at which CO_2_ emissions are sequestered is calculated in the following expression:

= (Binder Carbonation Rate/100*0.135*Binder Consumed Yearly)/Binder Concrete Service Life

**Figure 9:**
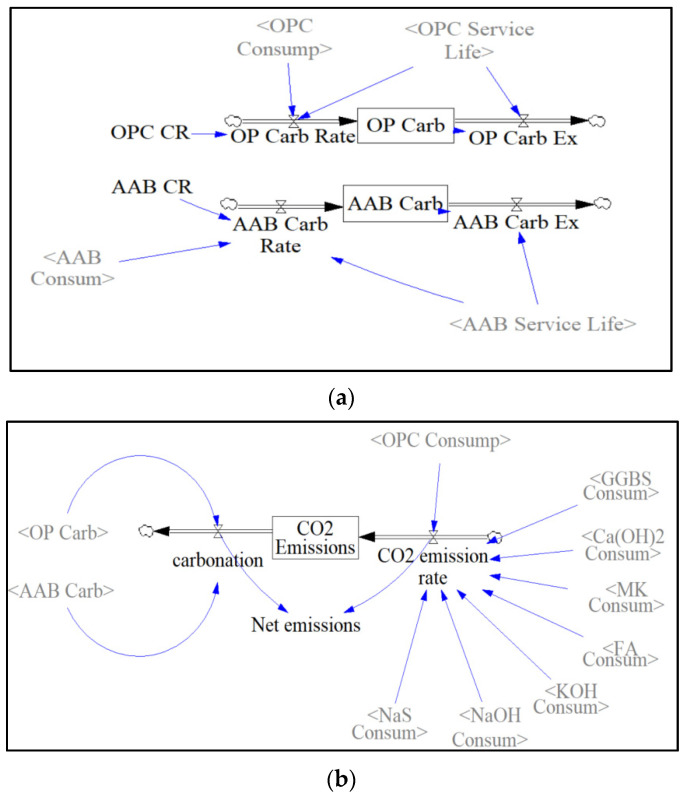
(**a**) Carbonation Sector; (**b**) CO_2_ Emissions Sector.

The amount of CO_2_ emissions sequestered over the 30-year period is later stored in the binder’s respective stock, as shown in Figure 9a. This accumulated amount is then carried out using the binder concrete service life as the transit time in a DELAY function. The net emissions are calculated by subtracting the total amount of CO_2_ emissions sequestered by carbonation from the CO_2_ emissions released every year. The CO_2_ emissions sequestered by carbonation are calculated by adding the two stocks, “AAB Carbonation” and “OBC Carbonation”, in the carbonation sector. Since net emissions are represented as an auxiliary block in Figure 9b, they are calculated on an annual basis, while the stock “CO_2_ emissions” represents the accumulated CO_2_ over the entire simulation period.

## 6. Model Sensitivity Analysis and Simulation Results

Sensitivity analysis with reference to the base case of OPC consuming 100% of the cement market was conducted to calibrate the Vensim model, ensuring the accuracy and validity of the results obtained from each simulation. The effects of key parameters: (1) Precursor type; (2) Activator type; (3) Policy implementation period; (4) Total AAB market share; (5) AAB carbonation rate; and (6) Service life of AAB concrete, were examined. The cases chosen for the sensitivity analysis of each parameter do not represent practical scenarios of experimental procedures conducted in previous research, as the main purpose behind the sensitivity analysis revolves around the reality check of the model presented. The effect of the precursor is examined by using an AAB mixture, assumed to comprise 70% precursor and 30% activator with a ratio of 0.42, as in most research studies. The activator is made of 50% NaOH and 50% Na_2_SiO_3_, the two most used alkaline solutions.

**Figure 10:**
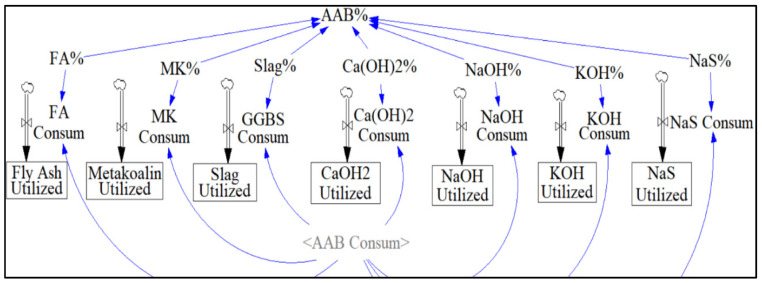
AAB Composition Sector.

The sector “AAB consumption” allows the user to observe the effect of AAB mixture proportion on net emissions (Figure 10). Since the Vensim model allows changing of the activator and precursor content using the sliding input device, a range of 50–80% of precursor and 20–50% of activator can be simulated using a slider feature built into the model.

**Figure 11:**
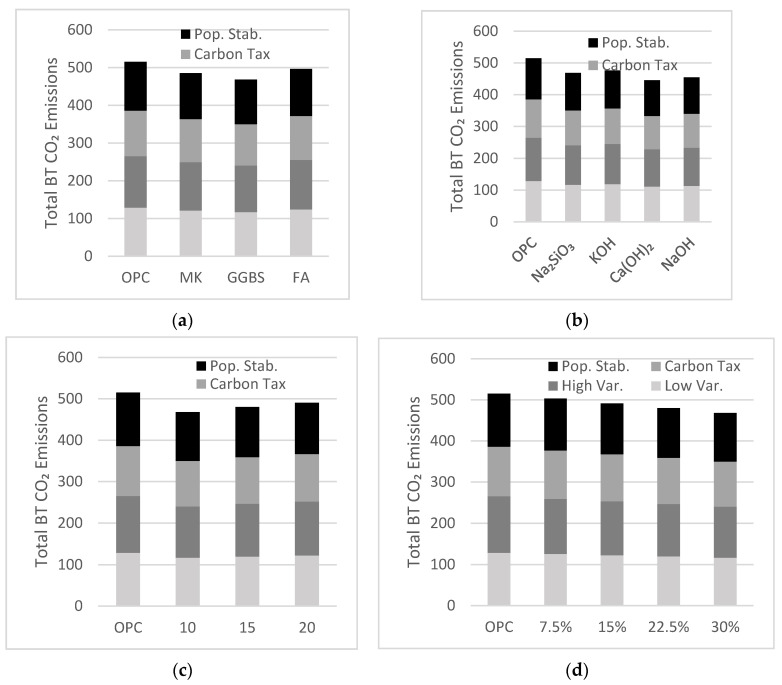
Sensitivity analysis exhibiting the effect of the (**a**) precursor type; (**b**) activator type; (**c**) policy implementation period; (**d**) AAB target market share demand in a Low Variability Scenario, High Variability Scenario, Carbon Tax Increase Scenario, Population Stabilization Scenario in the year 2050.

The simulation results shown in Figure 11a indicate the effect of each precursor on the total amount of CO_2_ emissions released at the end of the simulation period. GGBS was the preferable precursor in all scenarios, reducing the amount of net emissions by about 10% in year 2050 owing to its lower amount of CO_2_ emissions per ton. FA was the least favorable precursor as it only saved around 4% of net emissions by year 2050.

The effect of activator is studied using AAB mixtures, assumed to consist of 75% precursor and 25% activator, a design derived by Bernal et al. [27]. The simulations include alternative activators with reduced emissions (Figure 11b). The use of KOH and Na_2_SiO_3_ as activators reduced total net emissions by 7% and by 8–9%, respectively. Using Ca(OH)_2_ and NaOH as activators better reduced annual net emissions by up to 14 and 12%, respectively. Since the production of one ton of KOH releases about 1.94 ton of CO_2_, the projected reduction of annual net emissions is lower compared to that of other less emission-intensive alkaline activators. Although Ca(OH)_2_ can be used to effectively reduce net emissions, the type of precursor in an AAB mixture must be compatible with the type of activator. For example, because GGBS is rich in calcium and magnesium, the use of Ca(OH)_2_ is redundant and could lead to a binder with lower durability.

Cases for the policy implementation period are tested with a fixed market share of 30% and an AAB mixture made with 70% GGBS and 30% activator compromising 50% NaOH and 50% Na_2_SiO_3_. As mentioned previously, policy implementation periods of 10, 15, and 20 years were explored in this part of the sensitivity analysis. Net emissions decreased as the policy implementation period decreased (Figure 11c). A 20-year policy implementation period reduced net emissions by 5%, whereas a 10-year implementation period led to 10% total emission reduction. The sooner AABs gain momentum and market share, the more effectively they reduce GHG emissions.

Cases for the influence of AAB market share were tested with the policy implementation period fixed at 10 years and the same AAB mixture used for analyzing the effect of the policy implementation period. As the AAB market share increased, net emissions decreased (Figure 11d). Market shares of 7.5% and 30% reduced net emissions by 3%, and 8%, respectively. The use of AAB increased exponentially as per an open loop system where AAB production can spark further interest, causing more production of AAB.

**Figure 12:**
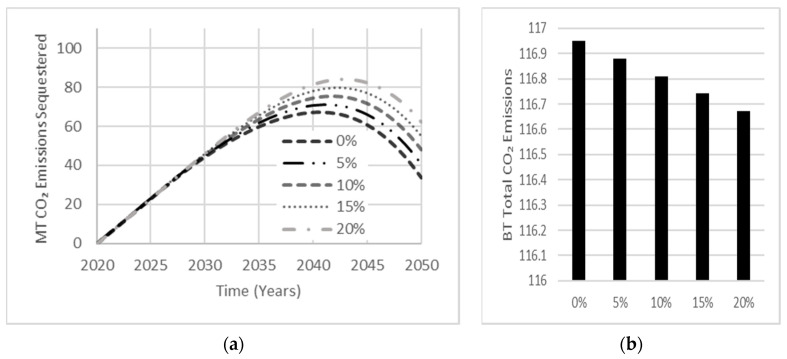
Sensitivity analysis exhibiting the effect of AAB carbonation rate on (**a**) total carbonation and, (**b**) total CO_2_ emissions released by the year 2050.

Sensitivity analysis conducted on the effects of AAB carbonation rate and service life indicated little to no influence on net emissions. Carbonation typically affects a small amount of concrete over the simulation period of 30 years. Moreover, the effect of service life over 30 years is minimal but would become more significant if the simulation period was extended. Figure 12 shows that increasing AAB carbonation increased the total amount of CO_2_ emissions sequestered over the simulation period, which peaks near the end of the simulation period, indicating a relationship between the amount of concrete produced and the quantity of CO_2_ emissions sequestered. With this relationship being established, the carbonation rate used for OPC base cases shall be set to an assumed carbonation percentage of 10% [12]. Since AAB concrete samples containing GGBS as the precursor have shown double the carbonation depth examined in OPC concrete samples [27], an assumed carbonation percentage of 20% shall be used for all AAB cases.

**Figure 13:**
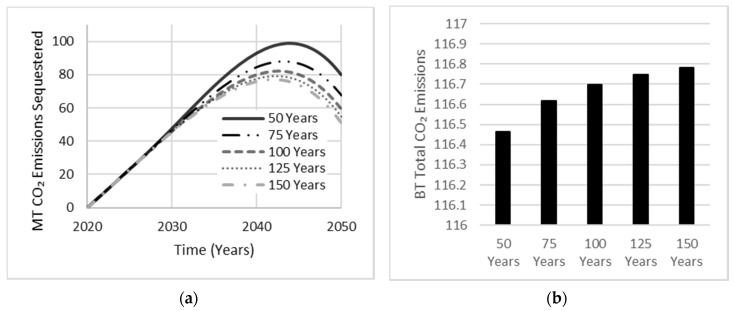
Sensitivity analysis exhibiting the effect of AAB service life on (**a**) total carbonation and (**b**) total CO_2_ emissions released.

As the AAB market share escalates to 30%, the annual production of OPC concrete will eventually stabilize, reaching a target goal of 70% market share. Hence, a depreciation in the amount of OPC concrete correlates to a depreciation in the total amount of CO_2_ emissions being sequestered, with OPC concrete sharing a larger portfolio with an assumed service life of 45 years by Nehdi et al. [12]. As the service life of AAB increased, the total amount of CO_2_ emissions sequestered decreased (Figure 13). Since this relationship suggests a more conservative approach in approximating the total amount of CO_2_ emissions released, an assumed service life of 90 years shall be used in all AAB cases. Although carbonation is known to decrease the durability of concrete structures via corrosion of steel reinforcement, leading to decreased service life, AABs have continuously demonstrated superior engineering characteristics when examining mechanical strength, thermal and durability properties of concrete [28].

## 7. Exploring Optimal AAB Cases

### 7.1. Single Precursor Systems

**Figure 14:**
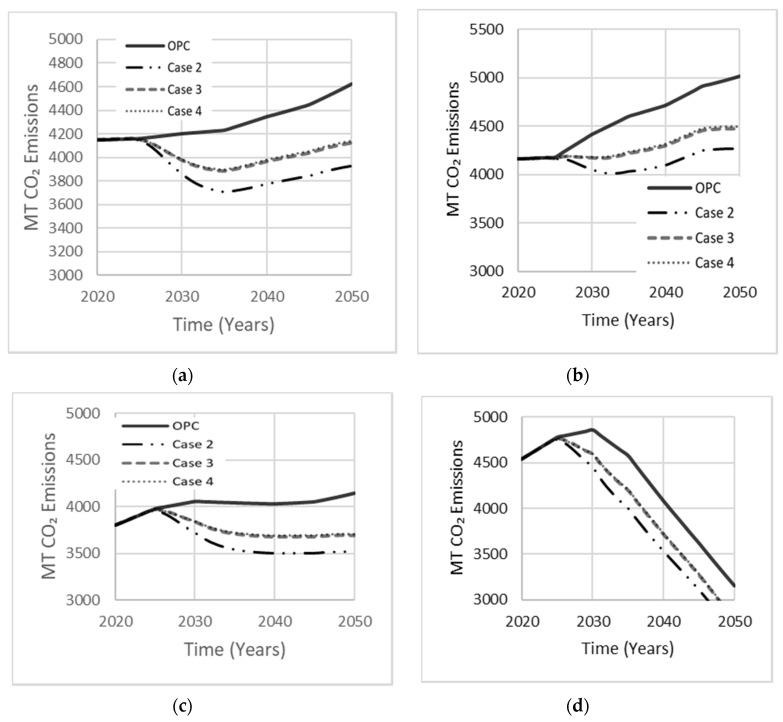
The effect of the single precursor system on net emissions released in (**a**) Low Variability Scenario; (**b**) High Variability Scenario; (**c**) Carbon Tax Increase Scenario; (**d**) Population Stabilization Scenario.

The composition of AAB can vary by proportioning the activator to precursor ratio to achieve optimal mechanical strength and durability. Since MK needs a relatively larger amount of alkali-activator due to its low Si:Al ratio, the MK dosage is usually 55–65%. The dosage of GGBS and FA is in the 70–80% range (Table 1). Each simulation case involves a single precursor with varying activator dosage, comprised of either Na_2_SiO_3_ or NaOH. Case 1 is a base scenario in which OPC occupies 100% of the total market. Case 2 was derived by Bernal et al. [29] using GGBS with Na_2_SiO_3_, which proved to achieve a higher mechanical strength of approximately 75 MPa with a 90-day curing period. Kim et al. [15] used LCA to model the CO_2_ emissions released by the AAB mixture examined in case 3. Fernandez-Jimenez et al. [30] reported an optimal compressive strength of 70 MPa for an AAB concrete made with FA precursor and NaOH as an activator, deriving the composition of case 4. Simulation results for these four cases are shown in Figure 14. The drop in net emissions observed in all cases at about ten years into the simulation period indicates increasing interest in AABs concrete, which started to gain market share. Case 2 produced least net emissions (16% reduction). Cases 3 and 4 reduced net emissions by comparable amounts but were less effective than case 2.

### 7.2. Single Precursor Systems with Hybrid Activators

**Figure 15:**
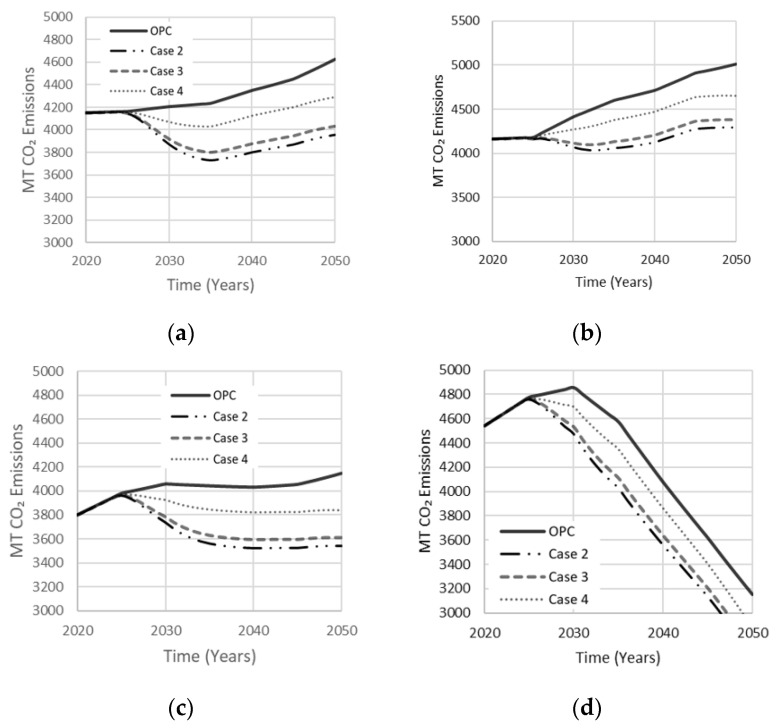
Effect of the single precursor system with optimal hybrid activator mixes on net emissions released in (**a**) Low Variability Scenario; (**b**) High Variability Scenario; (**c**) Carbon Tax Increase Scenario; (**d**) Population Stabilization Scenario.

Table 2 adopts hybrid activator mixtures with a single precursor. Case 2 was studied by Bernal et al. (2012), where a combination of NaOH and Na_2_SiO_3_ was used to activate the GGBS to produce optimal compressive strength of 78 MPa at a curing period of 90 days. A combination of NaOH and Na_2_SiO_3_ is best used with GGBS precursors because the framework structure of the resulting gel is ordered as a network rather than a chain [17]. Case 3 represents the simplest AAB mixture used in producing a co-existence of N-A-S-H and C-A-S-H gels to enhance compressive strength, using MK, Ca(OH)_2_, and NaOH [17]. The mixture represented in case 3 was derived by Alonso et al. [31], where MK and Ca (OH)_2_ were used at a 1:1 ratio with NaOH of 10% AAB to produce a strength of 30 MPa. Case 4 is a mixture resulting in an optimal compressive strength of 78 MPa reported by Pan et al. [32], where a hybrid activator of NaOH and Na_2_SiO_3_ was used with a FA precursor to reduce permeability and mitigate efflorescence. Simulation results are shown in Figure 15. Case 4 produced less reduction in net emissions, while cases 2 and 3 reduced net emissions by 15% and 14%, respectively, in agreement with the sensitivity analysis discussed earlier.

### 7.3. Binary Precursor Systems

**Figure 16:**
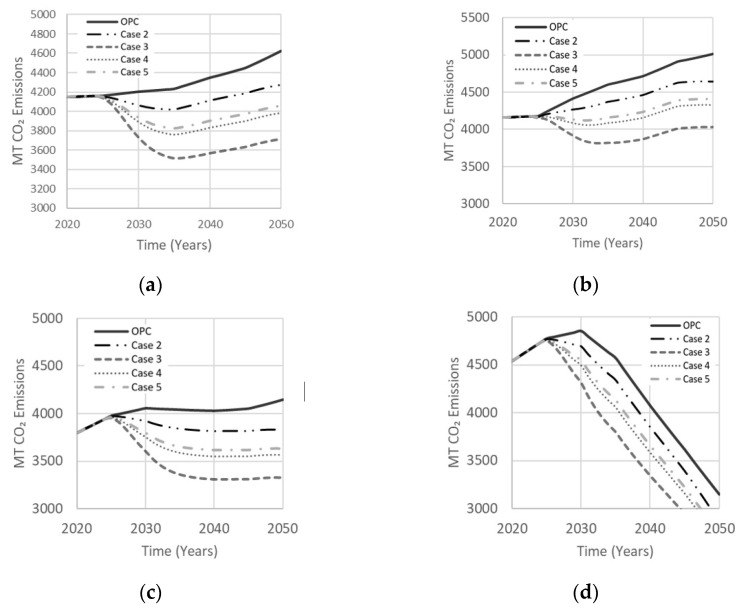
Effect of the binary precursor system on net emissions in (**a**) Low Variability Scenario; (**b**) High Variability Scenario; (**c**) Carbon Tax Increase Scenario; (**d**) Population Stabilization Scenario.

Cases in Table 3 explore the effects of various binary precursor systems on net CO_2_ emissions. Case 2 is a FA–MK-based AAM with thermal curing that has lower permeability and efflorescence, proposed by Zhang et al. [33] to produce an optimal compressive strength of 85 MPa. Case 3 is a 50% metakaolin and 50% GGBS with NaOH alkaline solution derived from a LCA study of geopolymers by Habert et al. [19] to reduce CO_2_ emissions, while producing an optimal compressive strength of 30 MPa. Case 4 also involves a hybrid mixture of GGBS and MK proposed to achieve enhanced durability compared to case 3 with more than double the compressive strength [34]. Mixing low and high calcium precursors in case 4 can produce coexistence of geopolymer and CSH gels for higher compressive strength, better durability and less emissions [17]. Finally, case 5 is a combination of GGBS and FA precursors with combined NaOH and Na_2_SiO_3_ activators, derived by Rafeet et al. [35] to produce an optimal compressive strength of 77 MPa in a 28-day curing period, though FA with NaOH can create efflorescence in-situ [17]. Figure 16 illustrates the effect of binary precursor systems on net emissions. Case 3 resulted in less net emissions owing to prominent use of GGBS and MK, two of the lowest CO_2_ emission precursors, along with a smaller percentage of NaOH. Case 2 was the least effective in reducing net emissions due to a larger activator-to-precursor ratio being required to activate metakaolin. Cases 4 and 5 reduced net emissions by about 12–13%. Although case 4 led to a 13% reduction in net emissions compared to 19% for case 3, it can offer a more attractive option because of its enhanced durability. Case 5, which also has good durability, resulted in similar net emissions to case 4.

### 7.4. Idealistic AAB Test Cases

Using the above sensitivity analysis and simulation data, a set of idealistic and futuristic AAB cases can be formulated (Table 4). Although these cases do not exhibit ideal mechanical strength properties, an optimal amount of reduced CO_2_ emissions over a 30-year simulation period can be achieved while theoretically satisfying factors such as durability and sustainability outcomes. Cases 2 and 3 combine binary precursor, which reduced CO_2_ emissions by 13–14%. According to Scrivener et al. (2018), the amount of available GGBS and FA could take up 16% of OPC production by year 2050, making GGBS and FA desired precursors in AABs. Since case 2 features better strength and durability than case 3, the policy implementation period was set to 10 and 15 years for cases 2 and 3, respectively. The NaOH activator in case 3 was replaced with Ca (OH)_2_ to release less CO_2_ whilst enhancing durability by co-existence of N-A-S-H and C-A-S-H gels. Cases 2 and 3 were given a target market share of 22.5% considering available precursor and activator production. Case 4 represents a single precursor system containing GGBS and Na_2_SiO_3_, where the policy implementation period was fixed at 10 years, while the targeted market share was halved to meet precursor and activator consumption.

**Figure 17:**
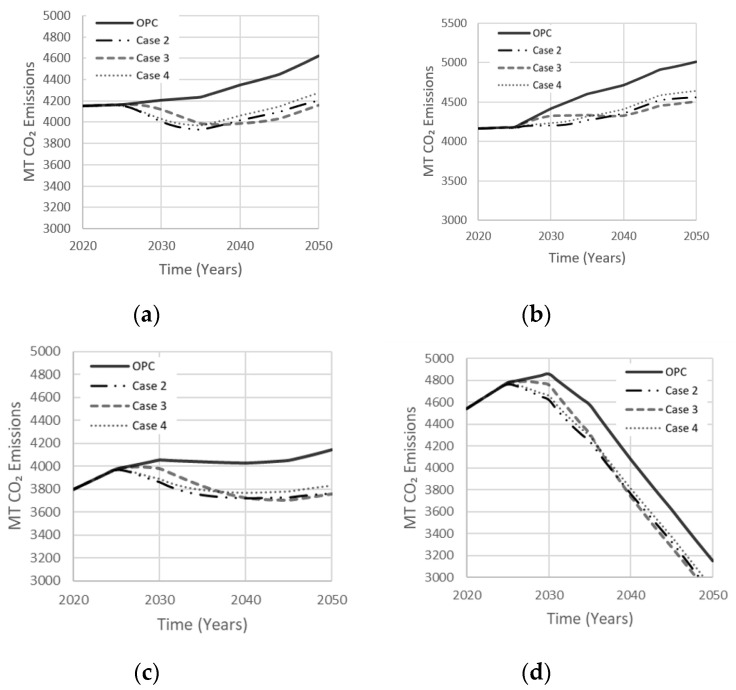
Effect of idealistic AAB cases on net emissions released in (**a**) Low Variability Scenario; (**b**) High Variability Scenario; (**c**) Carbon Tax Increase Scenario; (**d**) Population Stabilization Scenario.

**Figure 18:**
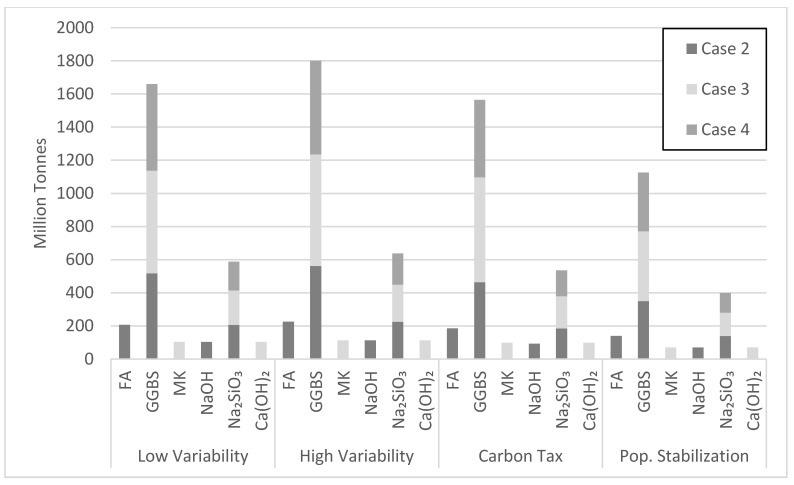
Projected amount of precursor and activator consumed in the year 2050.

Case 2 was most efficient in net emission reductions at the very beginning of the simulation period, followed by cases 4 and 3, respectively (Figure 17). Case 3 became more efficient around 16 to 18 years into the simulation due to its 5 year longer policy implementation period, resulting in a delay. Replacement of NaOH with Ca(OH)_2_ in case 3, which releases less CO_2_ emissions, also contributed to this drop. Case 3 first surpassed case 4 because of its larger targeted market share demand of 22.5% in comparison to 15% for case 4. The other scenarios in Figure 17 show similar trends to case 3 in reducing net emissions by the end of the simulation period, while surpassing cases 2 and 4 at various times, depending on the scenario. Although the test cases are implemented over different periods, they stabilize in a similar manner towards the end of the simulation period. Case 2 proved most efficient in reducing CO_2_ emissions in the long run. Its relatively large target market share and shorter policy implementation period contributed to this performance. The amount of FA consumed varied with the type of scenario (Figure 18). Since current production of FA is estimated at 900 million tons [14], the amount of FA consumed in the high variability scenario makes the tested scenario feasible, especially since countries with the largest OPC consumption are also the largest producers of FA. Figure 18 also shows the amount of GGBS consumed in various cement demand scenarios. With current production of GGBS at 330 million tons, the available GGBS should meet its projected demand through the year 2050. Therefore, scenarios based on GGBS appear feasible, though issues of logistics have not been duly explored herein.

The amount of Na_2_SiO_3_ activator needed to produce the proposed AAB seems to be a hurdle for making scenario 2 viable (Figure 18). The Na_2_SiO_3_ consumed in case 2 by the year 2050 is 140 million tons with a peak of 200 million tons in the year 2035. There are currently about 10 million tons of Na_2_SiO_3_ produced annually [14]. Thus, sizeable industry adjustments are needed to meet market demand. Figure 18 also captures the effect of the policy implementation time on Na_2_SiO_3_ consumption. Since cases 2 and 3 have similar target market share, the amount of Na_2_SiO_3_ consumed by 2050 will be similar. However, the 15-year policy implementation period for case 3 better allows Na_2_SiO_3_ production to grow by delaying the peak consumption towards the end of the simulation. The NaOH and Ca(OH)_2_ consumed during the simulation period do not seem to be a cause of concern (Figure 18). NaOH produced in 2015 was about 92 million tons, making case 2 feasible with the assumption of a robust increase in future production of NaOH. Moreover, since Ca(OH)_2_ is derived from limestone, an abundant amount of Ca(OH)_2_ could be readily available.

**Figure 19:**
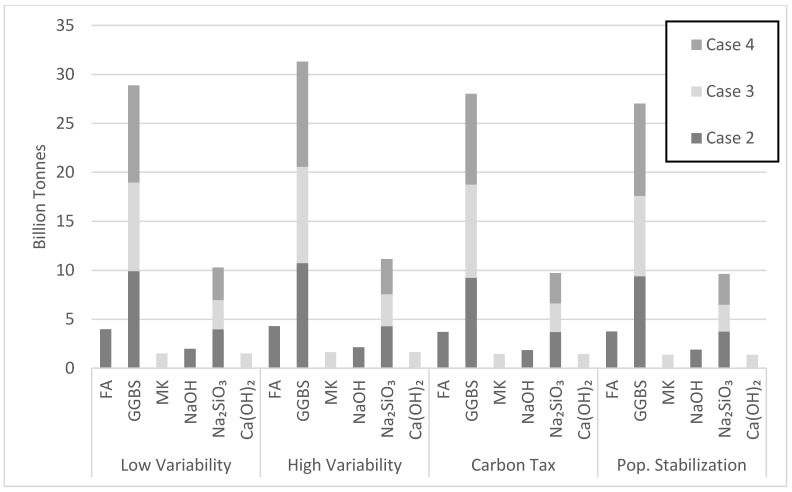
Total projected amount of precursor and activator utilized by the year 2050.

Case 3 requires large consumption of MK. Yet, Figure 18 points to issues with the projected MK consumption. The MK consumption in the high variability cement demand scenario by the year 2050 largely exceeds current production. Noting that metakaolin is used by various industry sectors, investments are needed to increase MK production over the next few decades. For instance, the state of Georgia in the USA has an estimated 10 billion tons of kaolinite clay, while Brazil has a deposit of 2 billion tons. Clearly, different viable scenarios of AAB production will emerge in different major cement producing countries depending on the available resources and local context. The largest amount of precursor used over the simulation period is GGBS at 10.74 billion tons in cases 2 and 4 for the high variability demand scenario (Figure 19). Although case 4 uses more GGBS than case 2, the amount of GGBS consumed in both cases was similar because of the 7.5% lower market share for case 2 (Figure 19). The largest amount of activator needed over the simulation period is Na_2_SiO_3_ at 4.3 billion tons in case 2. Na_2_SiO_3_ production seems to be a barrier for AABs, as pointed out earlier.

## 8. Model Extension and Needed Research

The proposed system dynamics model is not intended to predict future emissions. Given the structure of the system and its inherent assumptions, simulations can be used as guidance for policy making. The model can substitute for the linear thinking of the user to capture effects of time-dependent feedback and counterintuitive behavior. Hence, importance is given to the trends under certain policies and test scenarios, rather than the quantities of emission reductions. The user should be able to define the best strategies to implement over the next thirty years for using alternative binders in reducing GHG emissions from cement production. The dynamic approach provides flexibility to go beyond the borders of the model to accommodate other aspects such as economic growth, GDP gap, population growth or stabilization, etc. Rational assumptions were made for market share, policy delay periods, rate of carbonation and service life. However, accurate values from in-situ performance and market dynamics can sway the simulation results. Correlations between the various AABs and their carbonation, durability, mechanical strength, service life, etc. need to be better established in future research. Moreover, emissions released from raw materials extraction and transportation, as well as thermal curing, have not been included, but can be considered in future model enhancements. Nevertheless, reductions in CO_2_ emissions from cement production are possible through increased AAB market share. Enhanced concrete durability by AABs can also extend service life, reducing repair and replacement of concrete structures, thus further reducing emissions. There is clear need for developments of effective and reliable precursors and activators to meet the AABs market demand. Particular pressures on soda ash and kaolin reserves could be experienced to meet future demand for sodium silicate solutions and metakaolin, respectively. The construction sector will be more inclined to use AABs if protocols of the UN and governments for reducing GHG emissions are enforced, for instance by imposing carbon taxes. It should be noted that further sustainability benefits can be observed by coupling AABs with well-established high-volume replacement supplementary cementitious materials, better durability and sustainability design of concrete structures, substantial use of renewable energy sources in cement manufacturing, and implementing effective carbon capture, storage and utilization technologies in cement production.

## 9. Conclusions

This study uses systems thinking to model the effects of using alternative binders on future CO_2_ emissions from portland cement production. The novel model proposed is based on System dynamics and was built in a Vensim environment. It accounts for the type of AAM precursor and activator, AAM market share, durability and service life, and carbonation. Various scenarios for policy making were tested, including different projected cement consumption alternatives, single AAM precursor systems, single precursor systems with hybrid activators, and binary precursor systems. Based on the simulation results, the following conclusions can be drawn:

- If reliable and consistent sources of AAB precursors and activators become available and AAB technology becomes robust for large-scale implementation to gain significant construction market share, important CO_2_ emission reductions from cement production ranging from 10 to 20% could be achieved.
- The shorter the policy implementation period for AABs and the larger its potential market share, the more significant will be the net GHG emission reductions from cement production.
- Simulation results indicate that there is more performance and sustainability advantages in optimizing AABs with hybrid precursors and hybrid activators, than using single precursors.
- Among existing precursors, GGBS was found advantageous. It may be rational to exploit world production of GGBS in AAB systems, and dedicate fly ash, natural pozzolans, biomass and waste incineration ash, and other pozzolans to SCM applications.
- Simulation results show that to meet the policy intent for larger AAB market share, there will be pressure to produce more metakaolin as a precursor and Na_2_SiO_3_ as an activator.
- The novel model allows the testing of diverse policy scenarios, with inherent flexibility that permits coupling the model with economic and service life models. The modular architecture of the developed model also facilitates expanding its boundaries to capture other features of the holistic CO_2_ global emissions challenge.

## Figures and Tables

**Table 1:** Single precursor system.

Case	Binder Composition	Share of Total Concrete Demand (%)	Policy Implementation Time (Years)
**I**	Portland Cement	100	N/A
**II**	AAB: GGBS (75%) Na_2_SiO_3_ (25%)	30	10
**III**	AAB: MK (60%) NaOH (40%)	30	10
**IV**	AAB: FA (75%) NaOH (25%)	30	10

**Table 2:** Single precursor system with optimal hybrid activator mix.

Case	Binder Composition	Share of Total Concrete Demand (%)	Policy Implementation Time (Years)
**I**	Portland Cement	100	N/A
**II**	AAB: GGBS (70%) NaOH (10%) Na_2_SiO_3_ (20%)	30	10
**III**	AAB: MK (45%) Ca(OH)_2_ (45%) NaOH (10%)	30	10
**IV**	AAB: FA (70%) NaOH (15%) Na_2_SiO_3_ (15%)	30	10

**Table 3:** Binary precursor system.

Case	Binder Composition	Share of Total Concrete Demand (%)	Policy Implementation Time (Years)
**I**	Portland Cement	100	N/A
**II**	AAB: FA (10%) MK (55%) NaOH (10%) Na_2_SiO_3_ (25%)	30	10
**III**	AAB: MK (45%) GGBS (45%) NaOH (10%)	30	10
**IV**	AAB: MK (10%) GGBS (60%) NaOH (10%) Na_2_SiO_3_ (20%)	30	10
**V**	AAB: GGBS (50%) FA (20%) NaOH (10%) Na_2_SiO_3_ (20%)	30	10

**Table 4:** Feasible AAB mixtures for futuristic scenarios.

Case	Binder Composition	Share of Total Concrete Demand (%)	Policy Implementation Time (Years)
**I**	Portland Cement	100	N/A
**II**	AAB: GGBS (50%) FA (20%) NaOH (10%) Na_2_SiO_3_ (20%)	22.5	10
**III**	AAB: MK (10%) GGBS (60%) Ca(OH)_2_ (10%) Na_2_SiO_3_ (20%)	22.5	15
**IV**	AAB: GGBS (75%) Na_2_SiO_3_ (25%)	15	10
